# Comparison of Invasive Versus Non-Invasive Pulse Contour-Based Cardiac Output Measurements at Rest and During Exercise in Pulmonary Hypertension

**DOI:** 10.3390/jcm14248971

**Published:** 2025-12-18

**Authors:** Anna Titz, Julian Müller, Simon Raphael Schneider, Mona Lichtblau, Silvia Ulrich

**Affiliations:** 1 Department of Pulmonology, University Hospital Zurich, 8091 Zurich, Switzerland; anna.titz@usz.ch (A.T.); simonrafael.schneider@usz.ch (S.R.S.);; 2 Faculty of Medicine, University of Zurich, 8032 Zurich, Switzerland

**Keywords:** cardiac output, Modelflow, Finapres, thermodilution, direct fick, bland–altman, pulmonary hypertension, exercise, right heart catheterization

## Abstract

**Background/Objectives:** Measuring cardiac output (CO) is essential for diagnosis and therapeutic monitoring in pulmonary hypertension (PH). CO assessment based on thermodilution (TD) or Direct Fick (DF) during standard right heart catheterization (RHC) is impractical for regular follow-up. We evaluated the accuracy and agreement of non-invasive Modelflow (MF)-based CO assessment compared with TD and DF during rest and exercise RHC in PH. **Methods:** This post hoc analysis from a crossover RCT included 24 PH patients (7 females, 59 ± 14 years; mean pulmonary artery pressure 37 ± 11 mmHg) who underwent RHC with repetitive CO assessments at rest and during exercise. CO was measured by TD, DF, and non-invasive MF by fingertip pulse contour analysis at rest and during stepwise cycling to maximal exertion. **Results:** At rest, mean CO was comparable between methods: TD = 6.05 ± 1.80 L/min, DF = 5.68 ± 1.88 L/min, MF = 6.09 ± 1.84 L/min. At end-exercise, CO increased to TD = 11.18 ± 4.38 L/min, DF = 11.84 ± 4.74 L/min, MF = 8.38 ± 2.93 L/min. Bland–Altman showed minimal bias at rest (MF vs. TD: 0.04 L/min; MF vs. DF: −0.07 L/min) but substantial variability during exercise, with underestimation of CO by MF with increasing workloads (MF vs. TD bias = −2.80 L/min; MF vs. DF bias = −4.38 L/min). Limits of agreement were wide across all workloads. Linear regression confirmed an increasing CO with workload, but MF slope was shallower than TD/DF, suggesting proportional bias. Taffé analysis identified a significant differential (5.847) and proportional bias (0.195) indicative of CO overestimation by MF at low CO and underestimation at high CO. **Conclusions:** MF group-level agreement is acceptable, but individual-level accuracy is limited, indicating that MF may be suitable for trend monitoring but its applicability for clinical decision-making is restricted, especially during exercise.

## 1. Introduction

Pulmonary hypertension (PH) encompasses a spectrum of pulmonary vascular diseases, with pulmonary arterial hypertension (PAH) and chronic thromboembolic pulmonary hypertension (CTEPH) representing key subtypes characterized by progressive vascular remodeling, increased pulmonary vascular resistance (PVR), and increased risk for right ventricular failure [1]. Accurate hemodynamic assessment is essential for diagnosis, risk stratification, and therapeutic monitoring in these conditions, with cardiac output (CO) serving as a pivotal parameter for both initial evaluation and longitudinal follow-up [2,3,4].

Invasive measurement of CO remains the clinical standard and the choice of CO measurement method can impact the diagnosis and classification of PH as denominator to calculate PVR [5]. CO assessment by thermodilution (TD) via right heart catheterization (RHC) is widely used in adults. The Direct Fick (DF) method is considered the gold standard, particularly in the presence of intracardiac shunts or when thermodilution results are inconsistent, but requires equipment to simultaneously measure oxygen uptake [2,4,6]. Guidelines recommend these invasive techniques for diagnosis and invasive follow-up assessments noting their established accuracy in adult PH populations [1]. However, repeated invasive assessments are associated with procedural risks and patient discomfort. Therefore, non-invasive CO assessment techniques in chronic disease management at rest and during exercise would be desirable [2,4]. Previous studies have demonstrated beneficial results of non-invasive assessments, especially for patients with stable disease, those at increased risk from invasive procedures, and in the context of exercise testing to unmask early or latent disease [7,8,9,10]. Therefore, non-invasive measurement is particularly advantageous for patients with high procedural risk, those requiring serial monitoring, or when evaluating exercise physiology [1,5,11].

CO can be measured non-invasively using the Modelflow method, a waveform analysis for the beat-by-beat assessment that has been validated various times [3,12,13]. CO is reconstructed from finger arterial blood flow by simulating a three-element non-linear and time varying model of aortic compliance, a method validated before. Numerical integration of flow during systole provides stroke volume and CO is finally computed corresponding to the heart rate.

Nonetheless, systematic reviews indicate that non-invasive CO-techniques are not yet fully interchangeable with invasive methods, and their reliability may decrease at high CO values or in specific PH subgroups. Further validation and methodological refinement are needed to optimize their clinical utility [2,3,14]. We therefore analyzed data from a randomized controlled trial, where patients with PAH or CTEPH had repetitive CO measurements at rest and during exercise [15,16].

## 2. Materials and Methods

### 2.1. Study Design and Participants

**Figure 1:**
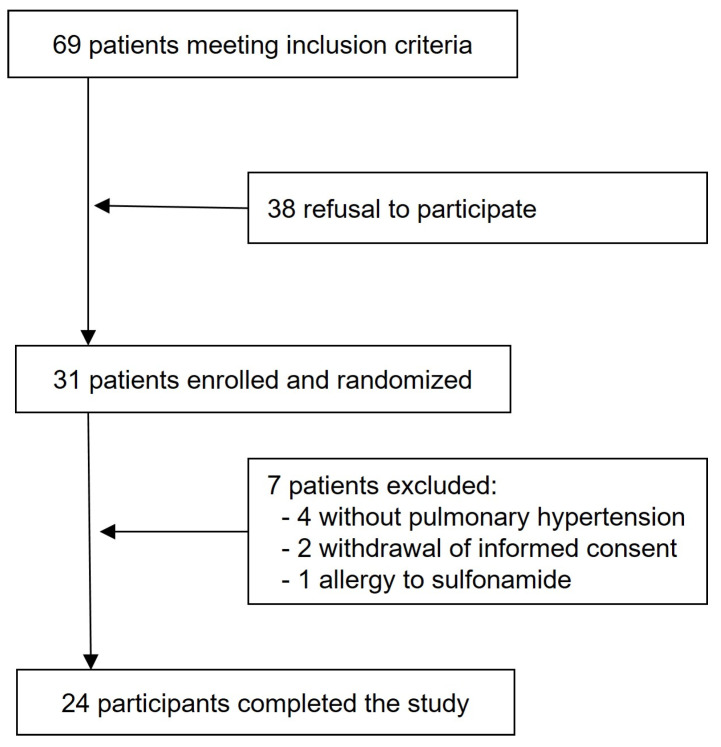
Study flow chart.

This is a post hoc analysis using data obtained during RHC at rest and during exercise during a prospective, randomized, placebo-controlled, double-blinded, triple-phase, crossover trial that evaluated the acute hemodynamic effects of acetazolamide vs. placebo (saline) at rest and during exercise in patients with PAH or CTEPH (Figure 1). In all patients, the RHC was clinically indicated, and written informed consent was obtained. The study complies with the declaration of Helsinki and was approved by the local ethics committee (BASEC 2016-00089), and the trial was registered at ClinicalTrials.gov (NCT02755259). The study design, methods, and results have been previously described, but non-invasively measured CO data have not been published before [15,16,17]. Patients performed cycling exercises in semi-supine position with 3 min stepwise incremental increase in work-rates by 10–20 Watts to maximal exhaustion (Thera-vital Ergometer; Medica Medizin GmbH, Lüdenscheid, Germany). Oxygen uptake (V’O_2_) was measured with a metabolic unit (Ergo-, Spirostik, Geratherm Respiratory, Bad Kissingen, Germany) and BlueCherry (Geratherm Respiratory, Germany) and averaged over 15 s.

### 2.2. Cardiac Output Measurements

CO was measured repetitively at rest and during stepwise exercise during three consecutive similar phases each 60–90 min apart according to the protocol. CO was measured using three different methods:

- Thermodilution (TD): CO was measured in triplicate by cold saline injection (Vigilance II, Edwards Lifesciences, Irvine, CA, USA) [18,19]. At each time point at rest and during each exercise step, two to three measurements were performed. Measurements were excluded if they varied >10% from each other, and the mean value was calculated using the remaining measurements.
- Direct Fick Method (DF): Arterial and mixed venous blood samples were taken from the radial artery line and the pulmonary artery catheter-tip in each phase at rest and end-exercise. To calculate the CO by the DF, we used the following formula: DF = V’O_2_ [L/min]/(hemoglobin [g/dL] × 13.4 × (SaO_2_-SmvO_2_ [%]/100) × 1000) [20].
- Continuous non-invasive hemodynamic measurement (MF): CO was measured continuously using the MF method, as described above, via the Finapres^®^ NOVA Basic device (Finapres Medical Systems, Enschede, The Netherlands) with the hemodynamics application module (including a finger cuff for CO measurements and an upper arm cuff for brachial calibration).

MF and other hemodynamic data were continuously measured and registered in LabChart (Version 8.1.16; ADInstruments, Dunedin, New Zealand), intermittently measured TD and DF were added to the system. LabChart data was thereafter inspected visually for plausibility, and artifacts were deleted and continuously registered data was extracted and averaged over 20 s intervals.

### 2.3. Statistical Analysis

Data are presented as mean ± SD unless indicated otherwise. Agreement between measurement methods was evaluated using Bland–Altman analysis, assessing bias and limits of agreement. As the Bland–Altman analysis does not account for possible heteroscedasticity, additionally, a Taffé analysis was performed to detect and quantify a proportional bias across the range of values, rather than assuming a constant bias [21]. Therefore, adjusted estimates of bias and proportional error across the range of CO were calculated. In addition, linear mixed regression analysis was performed to account for repeated measurements and determine the strength of correlation between the continuous non-invasive system and reference methods.

## 3. Results

### 3.1. Baseline Characteristics and Hemodynamic Measurements

A total of 24 patients (7 females) with PAH or CTEPH were included in the study. The mean age was 59 ± 14 years, and the mean pulmonary artery pressure was 37 ± 11 mmHg. Baseline demographic and hemodynamic data are summarized in Table 1.

CO measurements at rest and end-exercise, assessed by TD, DF, and MF, are presented in Table 2. At rest, mean CO values were 6.05 ± 1.80 L/min (TD), 5.68 ± 1.88 L/min (DF), and 6.09 ± 1.84 L/min (MF). At end-exercise, CO increased to 11.18 ± 4.38 L/min (TD), 11.84 ± 4.74 L/min (DF), and 8.38 ± 2.93 L/min (MF).

### 3.2. Comparison Between Measurement Methods

Table 3 shows the Bland–Altman analysis, which revealed variable agreement between the continuous non-invasive system and the reference methods.

When comparing MF vs. TD, the overall bias for CO was −1.39 L/min with limits of agreement (LoA) ranging from −7.34 to 4.57 L/min. Agreement was highest at rest (bias 0.04 L/min, LoA −4.09 to 4.17 L/min) and decreased at higher workloads (bias −2.80 L/min, LoA −11.57 to 5.98 L/min).

For MF vs. DF, the bias for CO was minimal at rest (–0.07 L/min, LoA −6.94 to 6.79 L/min) but, again, larger at maximal exercise (−4.38 L/min, LoA −16.18 to 7.43 L/min).

**Figure 2:**
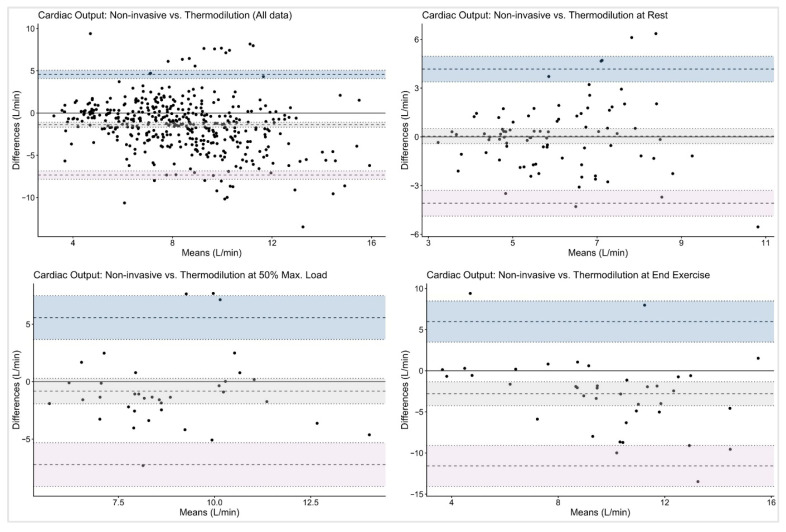
Bland–Altman plots on cardiac output with thermodilution as reference method. The given symbols represent the following: **•** individual subjects; ── bias (mean difference); ╍╍╍ limits of agreement; ┉┉┉ confidence interval for bias and limits of agreement.

**Figure 3:**
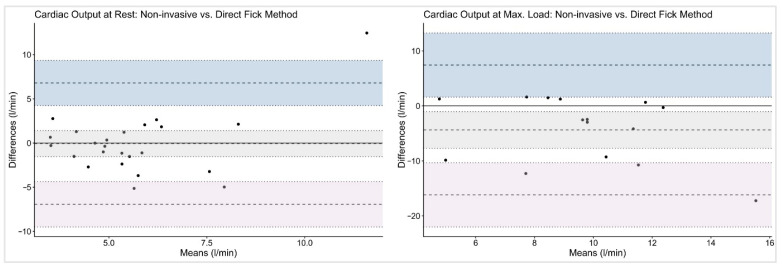
Bland–Altman plots on cardiac output with Direct Fick as reference method. The given symbols represent the following: **•** individual subjects; ── bias (mean difference); ╍╍╍ limits of agreement; ┉┉┉ confidence interval for bias and limits of agreement.

The graphical analyses illustrated the wide dispersion of CO values across all workloads and measurement methods. Bland–Altman plots (Figure 2 and Figure 3) revealed broad LoA between the non-invasive and reference techniques. The spread of data increased progressively with exercise intensity, accompanied by a tendency toward greater negative bias at maximal workloads.

**Figure 4:**
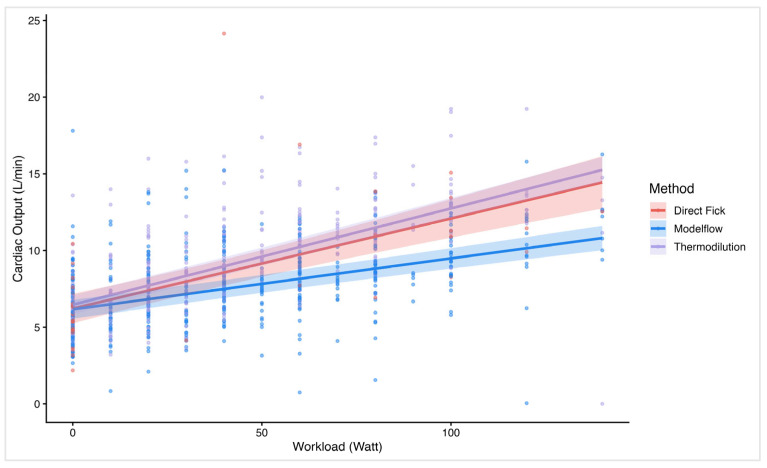
Linear regression plot on cardiac output over workload by method. The figure illustrates a linear regression line and individual data plots on cardiac output (*y*-axis) vs. workload (*x*-axis). Stratified by the different methods: Direct Fick, Modelflow, and thermodilution.

Linear mixed regression analysis confirmed significant correlations between workload and CO across all methods, which is in line with the findings of the Bland–Altman analyses. The plot (Figure 4) illustrates the relationship between workload and CO values measured by MF, TD, and DF. All methods show a positive correlation between CO and workload. Of interest, DF and TD show closely aligned slopes and intercepts, indicating better agreement and more accurate measurement. In contrast, the MF slope had a significantly flatter incline, suggesting a proportional bias and implying a systematic underestimation of CO measured by MF when compared to TD or DF.

**Figure 5:**
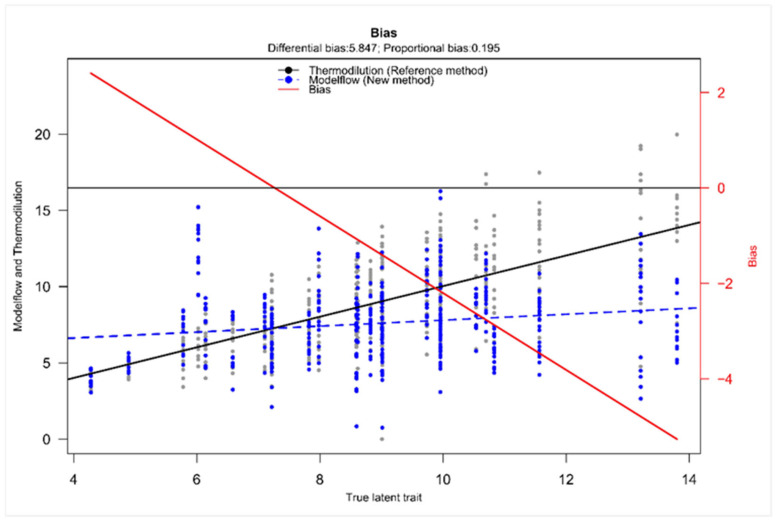
Taffé bias plot on cardiac output with thermodilution as reference method. The plot displays the estimated bias (difference between CO measured by MF or TD) as a function of the estimated true CO (given as true latent trait). The solid black line depicts the reference method’s measurement over the “true latent trait” (*x*-axis, given in L/min). The dashed blue line shows the new method. The red line indicates the bias (values are given at the right *x*-axis).

Taffé analysis was performed to further assess the systematic bias structure between TD and MF. Figure 5 visualizes the systematic difference between the two methods with a differential bias of 5.847. The proportional bias of 0.195 implied that the bias is not constant but changed depending on the magnitude of the true latent trait. The downward slope of the bias visualized the decrease in bias with increase in the true latent trait and therefore MF overestimates CO at low values and underestimates CO at higher values. The comparison plot emphasizes the findings of the bias plot, revealing a directionally dependent error with a non-uniform proportional pattern (Figure S1). Furthermore, the precision plot showed that TD remained the more precise method with a low and relatively constant standard deviation of measurement errors across the CO range, confirming stable and precise estimates regardless of the true latent trait. On the other hand, MF showed a higher dispersion of values, and the mean level of variability was substantially higher in MF compared to TD (Figure S1).

## 4. Discussion

This is a unique analysis of the accuracy of simultaneous non-invasive assessment of CO by pulse contour analysis by MF compared to the invasive DF and TD methods using large datasets of repetitive CO assessments during three phases of assessments each at rest and during stepwise cycling exercise RHC. We herein showed only a small mean bias of CO measured by the continuous non-invasive MF when compared with the reference methods. This indicates that, on average, the non-invasive MF technique did not systematically over- or underestimate CO across data points. However, despite this relatively minor bias, the limits of agreement were wide, reflecting considerable variability and limited agreement for individual measurements and substantial variability in individual measurements and limited precision, especially during dynamic states such as exercise or hemodynamic instability. Thus, overall, the MF-CO measurement is not suitable for individual patient decision.

Using Bland–Altman methods, we found that although mean bias between MF and the reference methods was small, both at rest and during stepwise cycling exercise, the wide limits of agreement preclude the use of this non-invasive method for individual patient decision-making at rest. These findings are consistent with previous validation studies comparing non-invasive pulse contour analysis with invasive reference methods. Previous studies also observed a small mean bias between Modelflow and reference CO measurements with minimal systematic over- or underestimation at the group level, indicating reasonable average agreement [3,22,23,24,25,26]. Accurate non-invasive measurements of CO would not only be highly appreciated at rest, but also during cardiopulmonary exercise testing, as often performed in the follow-up management of patients with PAH/CTEPH. However, in the present analysis, non-invasive MF limits of agreements even increased during exercise, which indicates substantial variability in CO estimation, particularly at higher outputs, and makes its use in clinical practice questionable (see Figure 2 and Figure 3).

As expected, all methods show a positive correlation between CO and workload, reflecting the physiological increase in CO with rising exercise intensity. While the mean bias is near 0 at rest when comparing MF with both TD and DF, the Bland–Altman analysis has shown relevant differences between methods at higher workloads and higher CO values, respectively. This suggests a proportional error that becomes more pronounced under exercise conditions. Of interest, TD and DF show closely aligned slopes and intercepts in the linear regression model, indicating better agreement and more accurate measurement. In contrast, the MF slope is significantly shallower, suggesting a proportional bias with an underestimation of values accentuated at higher workloads. Although the sample size of 24 participants is relatively small, the study’s repeated measures design yields a large number of data points, providing a solid foundation for the analysis and as the linear mixed model accounts for these repeated measurements, it strengthens the conclusion that systematic bias grows with workload.

While Bland–Altman analysis quantified the magnitude of the bias across the measurement spectrum, the Taffé analysis elucidates the pattern and direction of the bias and therefore serves as a valuable extension [21]. It demonstrated both a substantial differential bias and a proportional bias, emphasizing that the degree of underestimation by MF increased with higher values of the true latent trait. Moreover, the presence of a positive differential bias further indicates a systematic underestimation of MF values relative to the true latent trait. Even after accounting for the modeled true CO, MF continued to yield lower estimates, confirming a consistent downward bias and reinforcing the notion of systematic underestimation. As the Taffé method represents a distinct statistical concept from the Bland–Altman approach with a different analytical framework, this independently supports the findings.

Our findings are in line with previous Bland–Altman analyses, which have consistently shown that variability increases with higher cardiac outputs and during exercise, supporting the current observation of proportional error under these conditions [3,26]. This limits the reliability of single-point MF-based measurements for individual patient management, particularly in populations with pulmonary hypertension or during interventions requiring precise hemodynamic assessment [3,23,27]. Therefore, most recent reviews and meta-analyses conclude that while non-invasive pulse contour systems like Modelflow are suitable for trend monitoring, they are not interchangeable with invasive reference methods for clinical decision-making at the individual level [22,23,25,27]. While trending ability seems generally acceptable, absolute values should be interpreted with caution, especially in high-output or unstable states.

The proportional underestimation of MF-derived CO at higher workloads may reflect physiological and algorithmic limitations inherent to pulse contour analysis in PH. MF relies on waveform morphology to estimate stroke volume and CO. Decreased peripheral vasomotor tone leads to vasodilation, which has been shown to alter the arterial pressure waveform and reduce the amplitude and shape of the pulse contour, resulting in a less accurate calibration of the algorithm with underestimation of cardiac output [28]. This effect is exacerbated during exercise, where rapid changes in vascular tone and pressure are not fully captured by the model’s static assumptions and the inability to dynamically adjust for disease-related changes in compliance is a key limitation. MF’s algorithm assumes aortic compliance based on population averages, but in patients with PH, vascular stiffening and reduced compliance are common. This mismatch means the model does not accurately reflect the true pressure–volume relationship, especially at higher outputs, leading to systematic underestimation as CO rises [29]. Furthermore, PH itself alters right ventricular afterload and arterial impedance, potentially compounding measurement errors. Increased pulmonary vascular resistance and changes in systemic arterial impedance affect the transmission and reflection of pressure waves, which MF interprets to estimate flow. In addition, right ventricular dysfunction and elevated pulmonary pressures impair the ability of the right heart to augment output during exercise. This leads to abnormal pressure waveforms and further non-linearity in the pressure–flow relationship, compounding the underestimation by Modelflow at high workloads [29,30,31].

The results do not support the calculation of a uniform correction factor because the Taffé method analysis demonstrated a non-uniform, directionally dependent error: MF overestimated CO at low values and underestimated CO at higher values. This pattern indicates that a single linear correction factor would not adequately address the proportional bias across the full CO range. As the error varies depending on the output level, applying a uniform correction would risk introducing further inaccuracies at both extremes. Even though non-linear recalibration, as given by the Taffé method, could improve consistency and error structure by correcting the systematic bias and stabilizing measurement variability, this would only improve group-level accuracy, and, taking the wide limits of agreement into account, individual readings would still deviate too much for precise clinical use.

Our findings highlight that while MF-derived CO measurements may provide non-invasive trend data, its application for precise, individual-level hemodynamic assessment remains limited and should be discouraged. This study therefore highlights the need for individualized calibration or adaptive algorithms to improve accuracy across varying physiological states, particularly in disease populations with altered vascular mechanics.

## 5. Conclusions

In conclusion, the small mean bias with wide dispersion of data points observed across all workloads implies that while MF group-level agreement is acceptable, individual-level accuracy is highly limited. Consequently, while MF may be suitable for trend monitoring, its clinical applicability for precise hemodynamic assessment, particularly for decision-making in patients with PH, remains restricted. The broad limits of agreement highlight that single non-invasive CO measurements should be interpreted with caution, especially in dynamic or high-output states where measurement variability is greatest.

## Figures and Tables

**Table 1:** Baseline characteristics. Data are presented as mean ± SD unless indicated otherwise. PAH, pulmonary arterial hypertension; CTEPH, chronic thromboembolic pulmonary hypertension; NYHA, New York Heart Association; NT-proBNP, N-terminal pro B-type Natriuretic Peptide.

Baseline Characteristics		Number (%) or Mean ± SD
No. of patients		24
Sex	Male	17 (71%)
	Female	7 (29%)
Age, yr		59 ± 14
Body mass index, kg/m^2^		27.9 ± 4.6
Body surface area, m^2^		1.99 ± 0.19
Pulmonary hypertension classification	PAH	7 (29%)
	CTEPH	17 (71%)
NYHA class	I	2 (8%)
	II	15 (63%)
	III	7 (29%)
6 min walk distance, m		531 ± 112
NT-proBNP, ng/L (median [IQR])		258 [82, 485]
Heart rate, bpm		75 ± 11
SpO_2_, % (median [IQR])		93 [91, 95]
SmvO_2_, % (median [IQR])		63 [60, 67]
Mean pulmonary artery pressure, mmHg		37 ± 11
Pulmonary artery wedge pressure, mmHg		11 ± 2
Pulmonary vascular resistance, WU		5.2 ± 2.7
Mean systemic blood pressure, mmHg		94 ± 9
Right atrial pressure, mmHg		6 ± 3

**Table 2:** Hemodynamic measurements. Data are presented as mean ± SD unless indicated otherwise. *n* represents the number of valid data points included in the analysis.

Method	Cardiac Output at Rest (L/min)	*n*	Cardiac Output at End-Exercise (L/min)	*n*
Thermodilution	6.05 ± 1.80	83	11.18 ± 4.38	39
Direct Fick Method	5.68 ± 1.88	24	11.84 ± 4.74	15
Modelflow (non-invasive)	6.09 ± 1.84	83	8.38 ± 2.93	39

**Table 3:** Bland–Altman results on cardiac output measured by Modelflow compared to thermodilution and Direct Fick as reference method. Data are presented in L/min. LoA, Limit of Agreement. *n* represents the number of valid data points included in the analysis.

	Bias	Lower LoA	Upper LoA	Spread	*n*
Thermodilution					
All data	−1.39	−7.34	4.57	11.91	429
Rest	0.04	−4.09	4.17	8.26	83
All Loads	−1.72	−7.86	4.41	12.27	346
50% Max. Load	−0.82	−7.22	5.58	12.80	36
Max. Load	−2.80	−11.57	5.98	17.56	39
Direct Fick Method					
Rest	−0.07	−6.94	6.79	13.72	24
Max. Load	−4.38	−16.18	7.43	23.60	15

## Data Availability

Data are available upon request.

## Supplementary Materials

The following supporting information can be downloaded at: https://www.mdpi.com/article/10.3390/jcm14248971/s1. Figure S1: Taffé comparison plot and precision plot on cardiac output with thermodilution as reference method.

## Abbreviations

The following abbreviations are used in this manuscript:

CO	Cardiac output
CTEPH	Chronic thromboembolic pulmonary hypertension
DF	Direct Fick method
MF	Modelflow
PAH	Pulmonary arterial hypertension
PH	Pulmonary hypertension
PVR	Pulmonary vascular resistance
RHC	Right heart catheterization
SD	Standard deviation
TD	Thermodilution
V’O_2_	Oxygen uptake
