# Supplementary material for: Comparison of Invasive Versus Non-Invasive Pulse Contour-Based Cardiac Output Measurements at Rest and During Exercise in Pulmonary Hypertension
Source: J Clin Med. 2025 Dec 18;14(24):8971. doi: 10.3390/jcm14248971 (PMC12733934; doi:10.3390/jcm14248971)
Supplement: Supplementary file 1 [file jcm-14-08971-s001.zip › jcm-4041261-supplementary.pdf]

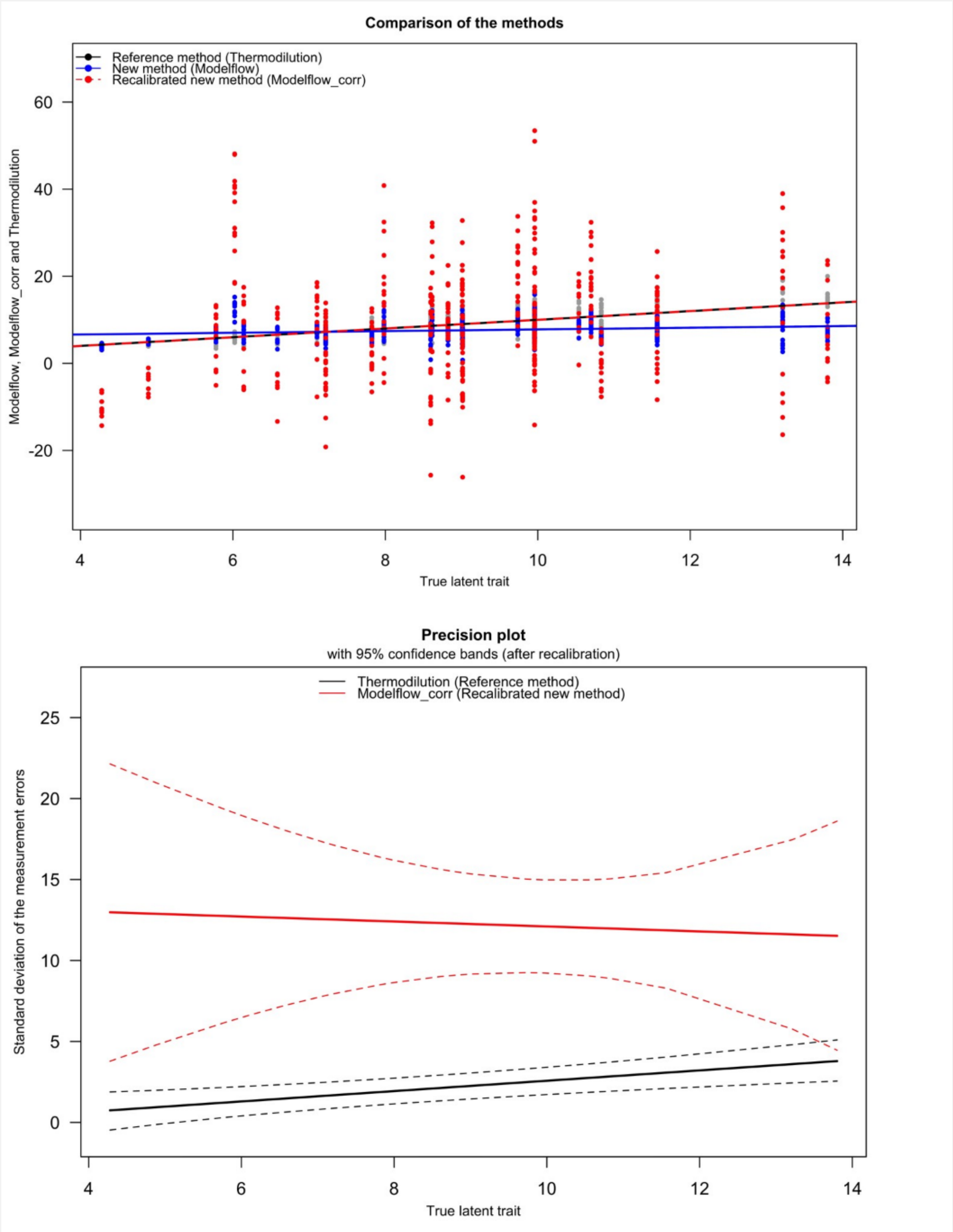

Figure S1. Taffé Comparison Plot and Precision Plot on Cardiac Output with Thermodilution as reference method. The comparison plot shows the relationship between the measurements retrieved by MF and TD as reference method with a regression line, revealing a directionally dependent error with a non-uniform proportional pattern. The precision plot illustrates the variability of the new method (MF) relative to the reference method (TD) displaying the standard deviation of the methods with its confidence intervals. The findings emphasize that TD remains the more precise method with the standard deviation of measurement errors remaining low and relatively constant across the CO range, confirming stable and precise estimates regardless of the true latent trait. On the other hand, MF shows a higher dispersion of values. However, the flat slope of the recalibrated MF (red line) shows a nearly uniform precision, even though the mean level of variability was substantially higher in MF compared to TD. The narrowing of the confidence bands toward mid-range values indicates an improvement in consistency and error structure after correction with recalibration correction of the systematic bias and stabilizing measurement variability. The "true latent trait" is given in l/min.
